# Economic Impact of a Hospital Cyberattack in a National Health System: Descriptive Case Study

**DOI:** 10.2196/41738

**Published:** 2023-06-30

**Authors:** Diana Portela, Diogo Nogueira-Leite, Rafael Almeida, Ricardo Cruz-Correia

**Affiliations:** 1 Department of Community Medicine, Information and Health Decision Sciences (MEDCIDS) Faculty of Medicine University of Porto Porto Portugal; 2 Doctoral Programme in Health Data Science (HEADS) Faculty of Medicine University of Porto Porto Portugal; 3 Nova School of Business and Economics Health Economics and Management Knowledge Center, New University of Lisbon Lisbon Portugal; 4 eMAIS: Movimento Associação dos Sistemas de Informação em Saúde Porto Portugal

**Keywords:** cybersecurity, medical informatics, economic impact, economic, cost, security, privacy, cyberattack, data breach, health system

## Abstract

**Background:**

Over the last decade, the frequency and size of cyberattacks in the health care industry have increased, ranging from breaches of processes or networks to encryption of files that restrict access to data. These attacks may have multiple consequences for patient safety, as they can, for example, target electronic health records, access to critical information, and support for critical systems, thereby causing delays in hospital activities. The effects of cybersecurity breaches are not only a threat to patients’ lives but also have financial consequences due to causing inactivity in health care systems. However, publicly available information on these incidents quantifying their impact is scarce.

**Objective:**

We aim, while using public domain data from Portugal, to (1) identify data breaches in the public national health system since 2017 and (2) measure the economic impact using a hypothesized scenario as a case study.

**Methods:**

We retrieved data from multiple national and local media sources on cybersecurity from 2017 until 2022 and built a timeline of attacks. In the absence of public information on cyberattacks, reported drops in activity were estimated using a hypothesized scenario for affected resources and percentages and duration of inactivity. Only direct costs were considered for estimates. Data for estimates were produced based on planned activity through the hospital contract program. We use sensitivity analysis to illustrate how a midlevel ransomware attack might impact health institutions’ daily costs (inferring a potential range of values based on assumptions). Given the heterogeneity of our included parameters, we also provide a tool for users to distinguish such impacts of different attacks on institutions according to different contract programs, served population size, and proportion of inactivity.

**Results:**

From 2017 to 2022, we were able to identify 6 incidents in Portuguese public hospitals using public domain data (there was 1 incident each year and 2 in 2018). Financial impacts were obtained from a cost point of view, where estimated values have a minimum-to-maximum range of €115,882.96 to €2,317,659.11 (a currency exchange rate of €1=US $1.0233 is applicable). Costs of this range and magnitude were inferred assuming different percentages of affected resources and with different numbers of working days while considering the costs of external consultation, hospitalization, and use of in- and outpatient clinics and emergency rooms, for a maximum of 5 working days.

**Conclusions:**

To enhance cybersecurity capabilities at hospitals, it is important to provide robust information to support decision-making. Our study provides valuable information and preliminary insights that can help health care organizations better understand the costs and risks associated with cyber threats and improve their cybersecurity strategies. Additionally, it demonstrates the importance of adopting effective preventive and reactive strategies, such as contingency plans, as well as enhanced investment in improving cybersecurity capabilities in this critical area while aiming to achieve cyber-resilience.

## Introduction

The delivery of health services has become increasingly digital, partially due to the introduction of information systems (ISs) [1]. Despite the focus on significantly reshaping the quality and efficiency of the provision of health care, the adoption of ISs did not come without risks [2-5].

Health care is an attractive target for cybercrime because of the wealth of personal data stored in hospital computer systems [6]. In the last decade, the frequency and size of cyberattacks in the health care industry have been rising, ranging from breaches of processes or networks to encryption of files that restrict access to data [2,7]. In 2017, the WannaCry international attack had an unprecedented scale; in the Portuguese national health system, it primarily affected the services of Hospital de Garcia da Orta (HGO) [8]. This attack was not directly targeted at any health institution, but it revealed how health systems worldwide are susceptible to cyber threats [8-11]. Cyberattacks are unique in the health field due to the type of information at risk and the consequences for patient safety from, for example, targeting electronic health records, access to critical information, and support for critical systems; cyberattacks can thereby cause delays in hospital activities, such as surgeries, drug delivery, and other treatments. These risks may paralyze health care systems, expose personal data of multiple stakeholders, reduce patient confidence, and ultimately threaten human life [12,13].

The effects of cybersecurity breaches are not only a threat to patients’ lives but also have financial consequences [3]. A single attack was estimated to have the potential to cost a hospital as much as US $7 million, which could lead to the long-term detrimental loss of reputation, activities, and revenue for hospitals and health facilities [3,14]. Given the enormous dependence on ISs, both for accessing and carrying out care, there is a growing difficulty in quantifying the true economic impact of these events. Moreover, while cybersecurity is critical to patient safety, it has historically been neglected. Regardless of cyber incidents being required to be reported and registered, the resulting data are not systematically processed or assessed, resulting in missed opportunities to understand vulnerabilities, risks, and threats [15,16]. Nonetheless, several media sources have highlighted the postponement of consultations, especially second consultations and continuity of care, as well as diagnostic tests and surgeries, due to the difficulty in accessing clinical data [17]. However, in a recent study on security incidents occurring globally during the COVID-19 pandemic, He et al [18] identified only 6 well-documented cyberattacks with detailed information available.

In this study, we aim, while using public domain data, to (1) identify data breaches in the Portuguese national health system since 2017 and, regarding the scarce availability of public information in Portugal, (2) measure the economic impact using an attack simulation on HGO as a case study.

## Methods

### Study Design

We retrieved information from multiple national media sources on cybersecurity from 2017 to 2022 using the keyword query “hospital” AND “cybersecurity/ciberseguranca” OR “cyberatacks/ciberataque” for each civic year. After building a timeline for the attacks in order to complement available information, we retrieved further information from local newspapers from the district where each attacked hospital was based. No restrictions based on publication date were applied.

Data were independently extracted from each source by 2 authors (DP and DNL) into a purposely built form that included the date of the attack; the type of attack; the duration and location of and a description of the attack; contingency plans; the return to normal activities; and media sources. A consensus was reached by the researchers on the absence of public information on each cyberattack in each civic year. In fact, health care use (namely, duration until full recovery, length of inactivity, or volume of inactivity as the result of each attack) was not reported in any public domain data. Thus, facing restricted or unavailable public domain data to obtain such information, we hypothesized the results of such attacks based on a case study (Textbox 1).

**Case study of a hospital cyberattack.**

A medium-large hospital care institution in Portugal (serving approximately 350,000 people) was struck by midlevel ransomware. This institution has a maturity level of III-IV according to the Healthcare Information and Management Systems Society framework. The ransomware infection is confirmed in the early hours of the day. The incident is detected by a user who reports to the information technology service that he or she no longer has access to all documents that are in the shared folders. At that moment, the prevention team starts tracking the problem. During this period, the number of application systems that are no longer operational grows until all management credentials are no longer valid, making it impossible for technical teams to check what is happening; following a domain controller reboot, a message appears informing the institution that it is suffering a ransomware attack. The institution’s contingency plan is activated due to the inoperability of the computer systems. The multidisciplinary response team is activated, whereby the institution’s technological partners are join in to carry out an exhaustive survey and report on the affected systems. As a result, all programmed activity is canceled and urgent activities are required to transition to paper forms. Patients from the emergency room are referred to other institutions, and authorities are notified. The institution uses a solution based on the lightweight directory access protocol (LDAP), which manages the different types of access per user and is integrated with all management solutions, including the backup solution, thus leading to slow progress. Regardless of this, a report is prepared to classify information based on the degree of criticality and difficulty of recovery, and a map of planned activities is made. During the first days, there is a reconstruction of backup infrastructure, access to repositories, and replacement of the LDAP solution, alongside the start of workstation cleaning. On day 9, the hospital is ready to resume the institution’s programed activity and reschedule all postponed activities. On day 21, the incident is closed, with the institution resuming 100% of scheduled activities.

Only direct costs were considered for estimates. Data for costs were retrieved according to predefined price and service levels as established in contract programs from the Central Administration of the Health System (Administração Central do Sistema de Saúde) [19]. The hospital program contract is an alternative to fee-for-service financing models where payment is made based on the number of procedures performed, regardless of the quality or outcome of the services provided. With the contract program, the focus shifts to delivering positive outcomes (considering production targets, accessibility, and quality) for the patient rather than just the quantity of procedures performed. This scenario used the HGO attack as a case study to represent both dimensions and services potentially affected; therefore, contract program values were used as assumptions. For an institution with the HGO characteristics, base values for yearly contract programs for each activity were retrieved considering external consultation (€22,700,338.00; a currency exchange rate of €1=US $1.0233 is applicable), hospitalization (€58,789,553.72), in- and outpatient clinics (€23,134,016.52 and €487,512, respectively), and emergency rooms (€10,771,535.29) [19].

The daily cost was based on the sum of the costs of all activities provided by the hospital in the case scenario. Since values are provided by year, we considered 250 working days for each year to calculate daily costs.

To ensure the reliability of the scenario, a sensitivity analysis was performed. While performing a sensitivity analysis, we aim to gain a better understanding of the potential range of economic impacts associated with a cyberattack for any extra day of activity lost and the volume of activity lost in each day. This involved testing the scenario under a range of different assumptions to assess the potential impact of variations in the assumptions on the estimated economic impact. We further assumed daily costs of inactivity as the percentage of services not provided by each day (25%, 50%, 75%, or 100% of total inactivity).

As the scenario should be tested under different assumptions (for example, using different contract programs or hospital dimensions), we also developed an open-source economic impact simulator.

### Ethical Considerations

This is a secondary data study that uses publicly available cost data; thus, there was no requirement for ethical approval.

## Results

**Figure 1:**
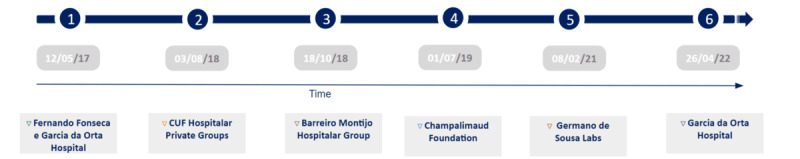
Incidents in hospitals in recent years.

Following the WannaCry cyberattack [8], from 2017 to 2022, we were able to identify 6 incidents (Figure 1) in Portuguese public hospitals using public domain data (there was 1 incident each year and 2 in 2018). Nevertheless, due to scarce information on such attacks concerning the type of activities (hospitalizations, surgeries, consultations, and loss of data) affected and the duration of inactivity, both overall and for each type of inactivity, we created a hypothetical scenario (see Methods section).

Hospital financing is based on contract programs, which differ according to the type of hospital. We used the HGO cyberattack as a reference study in order to base assumptions on costs and type of activities and to gather information on costs per type of inactivity [19], retrieve information on the population served by this institution [19], and define inactivity based on percentages of duration and dispersion of the attack along affected resources [20]. Thus, we estimated a daily cost impact, which highlights that, for an institution such as the HGO (serving a population of approximately 350,000 people), postponing 50% of external consultations (amounting to a yearly contract value of €22,700,338.00) may have a daily cost impact of €45,400.66 to the institution. To enhance the use and auditability of our research, we developed an open-source economic impact simulator for health care cyberattacks [21]. To better understand the impact of a given parameter in our simulator, a user can change only the parameter of interest and keep the remaining parameters at hypothetical baselines.

**Figure 2:**
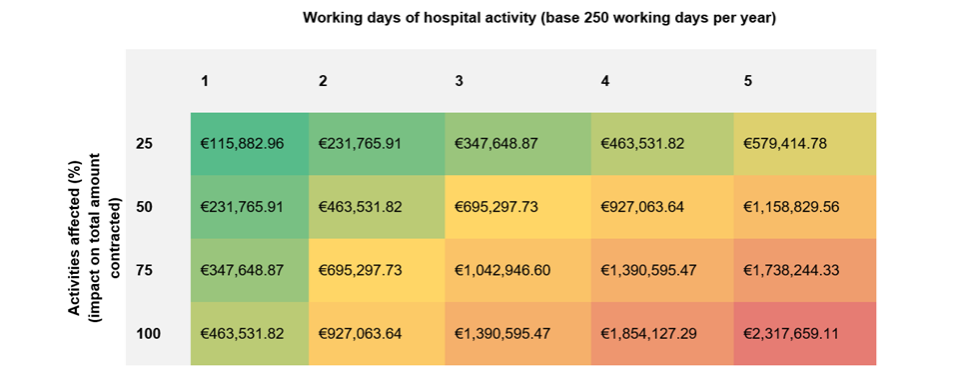
Sensitivity analysis for a cyberattack on a base contractual cost of €115,882,955.53 (considering external consultation, hospitalization, inand outpatient clinic, and emergency room). A currency exchange rate of €1=US $1.0233 is applicable.

Additionally, we performed a sensitivity analysis for both an optimistic and pessimistic scenario while considering the number of affected working days and the percentage of activities affected (Figure 2). Total costs can range between €115,882.96 and €2,317,659.11 depending on the expected minimum and maximum duration and percentage of impacted activities and working days (Figure 2). Results are provided for a working week (1-5 days of hospital activity). In the hypothesized scenario (of 9 potential days affected by the cyberattack), the estimated total cost ranges from €1,042,946.60 to €4,171,786.40 according to the volume of inactivity. In this scenario, until total recovery (at 21 days), this attack could cost between €2,433,542.07 and €9,734,168.26 according to the volume of affected activities.

## Discussion

Before the cyberattack on HGO that took place on April 26, 2022, it had been about 5 years since the first major cyberattack occurred on an institution of the National Health Service with such a scale (namely Fernando da Fonseca Hospital in 2017, in a sequel of the WannaCry cyberattack). This group of scientists was dedicated to identifying subsequent incidents since 2017 and estimating the potential economic impact of cyberattacks on the Portuguese health system. In the absence of public information on cyberattacks, reported drops in activity were estimated for the HGO using a hypothetical scenario of affected resources and percentages and duration of inactivity. This scenario was used to inform the cost assumptions, which were based on the dimensions of services described in the hospital case study, as this reflects the hospital financial method applied in the Portuguese health system.

We developed a model based only on available public domain data. Estimates were produced based on planned activity through the hospital contract program. We use sensitivity analysis to illustrate how midlevel ransomware might impact health institutions’ daily costs (inferring a potential range of values based on assumptions). Given the heterogeneity of our included parameters, we also provided a tool for users to distinguish such impacts of different attacks on institutions according to different contract programs, served population size, and proportion of inactivity.

As a result, financial impacts were obtained from a cost point of view, where estimated values have a minimum-to-maximum range of €115,882.96 to €2,317,659.11. Costs of this range and magnitude were inferred assuming different percentages of affected resources and with different numbers of working days while considering the costs of external consultation, hospitalization, in- and outpatient clinics, and emergency rooms for a maximum of 5 working days. Cybersecurity in the health sector is an essential part of maintaining user security, privacy, and trust in both the importance and the vital value of the information stored in such systems and the direct and indirect long-term impact on the quality of care and health outcomes. Thus, it must become part of the customer service culture and must be carefully planned [22]. The main challenges of cybersecurity in the health sector, as well as crucial health sector security solutions and areas for improvement, have been highlighted by He et al [18] and involve technology, processes, and people. Moreover, Jalali and Kaiser [3] emphasized the complexity of hospitals’ organizational models and their key stakeholders. To this end, they developed a model to consider the systemic effect of different characteristics on a single hospital’s ability to remain robust against cyber breaches; this suggested that different measures across hospitals can reduce the likelihood of cybercriminal attacks [3].

The risk and impact of cyberattacks increase as organizations become technologically more dependent and connected [23]. Quantifying the impacts of cyberattacks on health care organizations can be a challenge as many factors should be considered. Among these are limited data (as these data tend be self-reported, which can limit the accuracy of results), the methodologies used (whether they consider direct costs or both direct and indirect costs), the cost variation (as it depends on factors such as the type of attack, downtime, organization size, and value of compromised data), or the context (how these incidents affect the organization’s daily operations, patient safety, and public trust) [24,25]. Moreover, the ramifications of cyberattacks transcend economic impacts, and health outcomes should also be considered, which may be more difficult to measure and quantify compared to the financial costs of such incidents [23].

In this study, we were severely constrained by the lack of publicly available documentation on cyberattacks in the health sector in Portugal and their respective impacts on care services. This is the main reason why our cost analysis was extremely conservative, including neither indirect nor long-term costs (ie, quality of life or other indicators). A true economic approach that also encompassed benefits would require details currently unavailable to the public. Furthermore, our sensitivity analysis was based on the totality of services affected measured against the total value of the program contract, as there is no information on potentially asymmetric impacts depending on, for example, technological maturity or organization size.

In conclusion, regardless of the above-discussed limitations, our study provides valuable information and preliminary insights that can help health care organizations better understand the costs and risks associated with cyber threats and improve their cybersecurity strategies. Additionally, it demonstrates the importance of adopting effective preventive and reactive strategies, such as contingency plans, as well as enhanced investment in improving cybersecurity capabilities in this critical area while aiming to achieve cyber-resilience. It is our sincere hope that it kick-starts a much-needed discussion on the topic, one that can catalyze more accurate economic, organizational, and technical analyses in the future.

## Abbreviations

HGO: Hospital de Garcia da Orta
IS: information system

## References

[1] Laudon KC, Laudon JP (2020). Management Information Systems: Managing the Digital Firm, 16th edition.

[2] The Lancet Respiratory Medicine (2021). Digital health: balancing innovation and cybersecurity. Lancet Respir Med.

[3] Jalali MS, Kaiser JP (2018). Cybersecurity in hospitals: a systematic, organizational perspective. J Med Internet Res.

[4] Ghafur S, Grass E, Jennings NR, Darzi A (2019). The challenges of cybersecurity in health care: the UK National Health Service as a case study. Lancet Digit Health.

[5] Dönmez E, Kitapçı NS, Kitapçı OC, Yay M, Aksu PK, Köksal L, Mumcu G (2020). Readiness for health information technology is associated to information security in healthcare institutions. Acta Inform Med.

[6] No authors listed (2021). Cyberattacks cripple dozens of U.S. hospitals. Am J Nurs.

[7] Healthcare data breach statistics. The HIPAA Journal.

[8] (2022). Tentativas de ataque a hospitais de norte a sul continuaram mesmo após alerta no Garcia de Orta. Diário de Notícias.

[9] (2017). Piratas informáticos atacam hospital Garcia de Orta. SAPO.

[10] Maia A, Trigueirão S (2022). Hospital Garcia de Orta alvo de ataque informático. No Litoral Alentejano houve uma tentativa de ciberataque. Publico.

[11] SPMS (2017). Circular Normativa n.º 01—SPMS: Medidas excepcionais ciber-segurança. Serviços Partilhados do Ministério da Saúde.

[12] Portela D, Frade S, Patrício Patrícia, Cruz-Correia R (2022). Perspectives on the present and future of electronic health records in Portugal. Acta Med Port.

[13] Magnuson JA, Dixon BE (2020). Public Health Informatics and Information Systems.

[14] Claunch D, McMillan M (2013). Determining the right level for your IT security investment. Healthc Financ Manage.

[15] Zarocostas J (2021). Health under cyberattack. Lancet.

[16] Coventry L, Branley D (2018). Cybersecurity in healthcare: a narrative review of trends, threats and ways forward. Maturitas.

[17] Argaw ST, Troncoso-Pastoriza JR, Lacey D, Florin MV, Calcavecchia F, Anderson D, Burleson W, Vogel JM, O'Leary C, Eshaya-Chauvin B, Flahault A (2020). Cybersecurity of hospitals: discussing the challenges and working towards mitigating the risks. BMC Med Inform Decis Mak.

[18] He Y, Aliyu A, Evans M, Luo C (2021). Health care cybersecurity challenges and solutions under the climate of COVID-19: scoping review. J Med Internet Res.

[19] (2021). Acordo Modificativo ao Contrato-Programa 2021. ACSS.

[20] (2022). Reabertas urgências de obstretícia do Garcia de Orta após noite encerradas por escassez de médicos 2022. TSF.

[21] Economic impact simulator.

[22] Kosutic D (2021). The impact of cybersecurity on competitive advantage.

[23] Javaid M, Haleem A, Singh RP, Suman R (2023). Towards insighting cybersecurity for healthcare domains: a comprehensive review of recent practices and trends. Cyber Secur Appl.

[24] Renaud K, Coles-Kemp L (2022). Accessible and inclusive cyber security: a nuanced and complex challenge. SN Comput Sci.

[25] Wasserman L, Wasserman Y (2022). Hospital cybersecurity risks and gaps: review (for the non-cyber professional). Front Digit Health.

[26] PhD Programme in Health Data Science. HEADS.

